# A Case Report of Preoperative Stereotactic Body Radiotherapy as Part of Multimodal Treatment in Pediatric Extraskeletal Ewing Sarcoma: An Innovative Treatment Strategy

**DOI:** 10.7759/cureus.106872

**Published:** 2026-04-12

**Authors:** Sepideh Mohammadipour, Melis Gultekin, Hilal Susam Sen, Burak Ardicli, Mustafa Gokhan Gedikoglu, Diclehan Orhan, Berna Oguz, Murat Fani Bozkurt, Ali Varan, Ferah Yildiz

**Affiliations:** 1 Radiation Oncology, Hacettepe University Faculty of Medicine, Ankara, TUR; 2 Pediatric Oncology, Hacettepe University Faculty of Medicine, Ankara, TUR; 3 Pediatric Surgery, Hacettepe University Faculty of Medicine, Ankara, TUR; 4 Pathology, Hacettepe University Faculty of Medicine, Ankara, TUR; 5 Radiology, Hacettepe University Faculty of Medicine, Ankara, TUR; 6 Nuclear Medicine, Hacettepe University Faculty of Medicine, Ankara, TUR

**Keywords:** ewing sarcoma, extraosseous ewing sarcoma, pediatric ewing sarcoma, preoperative radiation therapy, sbrt (stereotactic body radiotherapy)

## Abstract

Extraosseous or extraskeletal Ewing sarcoma (E-EWS) is a rare condition that is best managed with a multidisciplinary approach. Radiotherapy (RT) constitutes a cornerstone in the treatment of the EWS tumor group and serves as neoadjuvant or adjuvant therapy, as well as definitive local treatment. Limited information exists on preoperative treatment options for this tumor entity, including stereotactic ablative RT, particularly in the pediatric population. This case report describes a nine-year-old girl with E-EWS located at the left lower costal margin who was successfully treated with multimodal therapy, including preoperative RT.

## Introduction

Extraosseous or extraskeletal Ewing sarcoma (E-EWS), characterized by small, round, blue cells, is a member of the EWS family of tumors [1]. It accounts for nearly 20% of EWS cases and typically originates in the soft tissues of the trunk and extremities [2,3]. It generally carries a more favorable prognosis than bone-derived cases [4]. In the meta-analysis published by Ghandour et al., the five-year overall survival (OS) for this population was 69%, with recurrence and distant metastasis occurring in 35% and 16% of cases, respectively [1].

The current management strategy for E-EWS, although controversial due to its rarity, generally requires a multidisciplinary approach, including systemic multi-agent chemotherapy, surgery, and radiotherapy (RT) [1,4]. RT plays a critical role in the treatment of EWS, whether in the neoadjuvant or adjuvant setting or as definitive local treatment in unresectable primary tumors despite induction chemotherapy [5].

Postoperative RT improves local control (LC) and event-free survival (EFS) in patients with high-risk factors for recurrence [6], but data on preoperative RT are limited. Generally, in large tumors, marginally resectable tumors, and inadequate clinical response to induction chemotherapy, preoperative RT is considered due to several theoretical advantages, such as minimizing the volume of normal tissue exposed to radiation, increasing radiosensitivity as a consequence of enhanced tumor oxygenation before resection, and facilitating removal of irradiated tissue during surgery [5,7]. Despite its advantages, preoperative RT is not widely used, mainly due to concerns about increased surgical complications and wound healing problems [8].

Preoperative RT in EWS is usually administered at conventional doses; however, if an R0 resection is not achieved, additional doses may be required [7]. Recently, hypofractionated RT schemes have become popular for preoperative sarcoma treatment [8]. Short-course RT, which reduces treatment time, helps avoid delays in surgery or systemic therapy, boosting patient compliance and lowering costs.

Local ablative treatments such as stereotactic body RT (SBRT) or ablative RT (SABR), with higher fraction sizes, offer high LC rates and low toxicity. SBRT in pediatric patients mostly applies to metastatic and recurrent cases, with limited data on safety and effectiveness for primary tumors [9]. The LITE-SABR meta-analysis has highlighted the growing attention toward SBRT in pediatric, adolescent, and young adult patients [10]. In this study, which included 30.2% (43/142) of patients with EWS, the one- and two-year LC rates were around 83.5% and 74%, respectively, with low serious toxicity (~2.9%). The present paper highlights a successful case of preoperative SBRT in E-EWS.

## Case presentation

A nine-year-old girl presented to the hospital with complaints of noticeable swelling and pain in her back. The ultrasound examination revealed a heterogeneous, hypoechoic soft tissue mass with a nodular component showing increased vascularity, located under the skin on the left side of the back, near the lower rib cage. With a preliminary diagnosis of soft tissue sarcoma, she was referred to the pediatric oncology department of our hospital, which is a tertiary cancer center.

**Figure 1:**
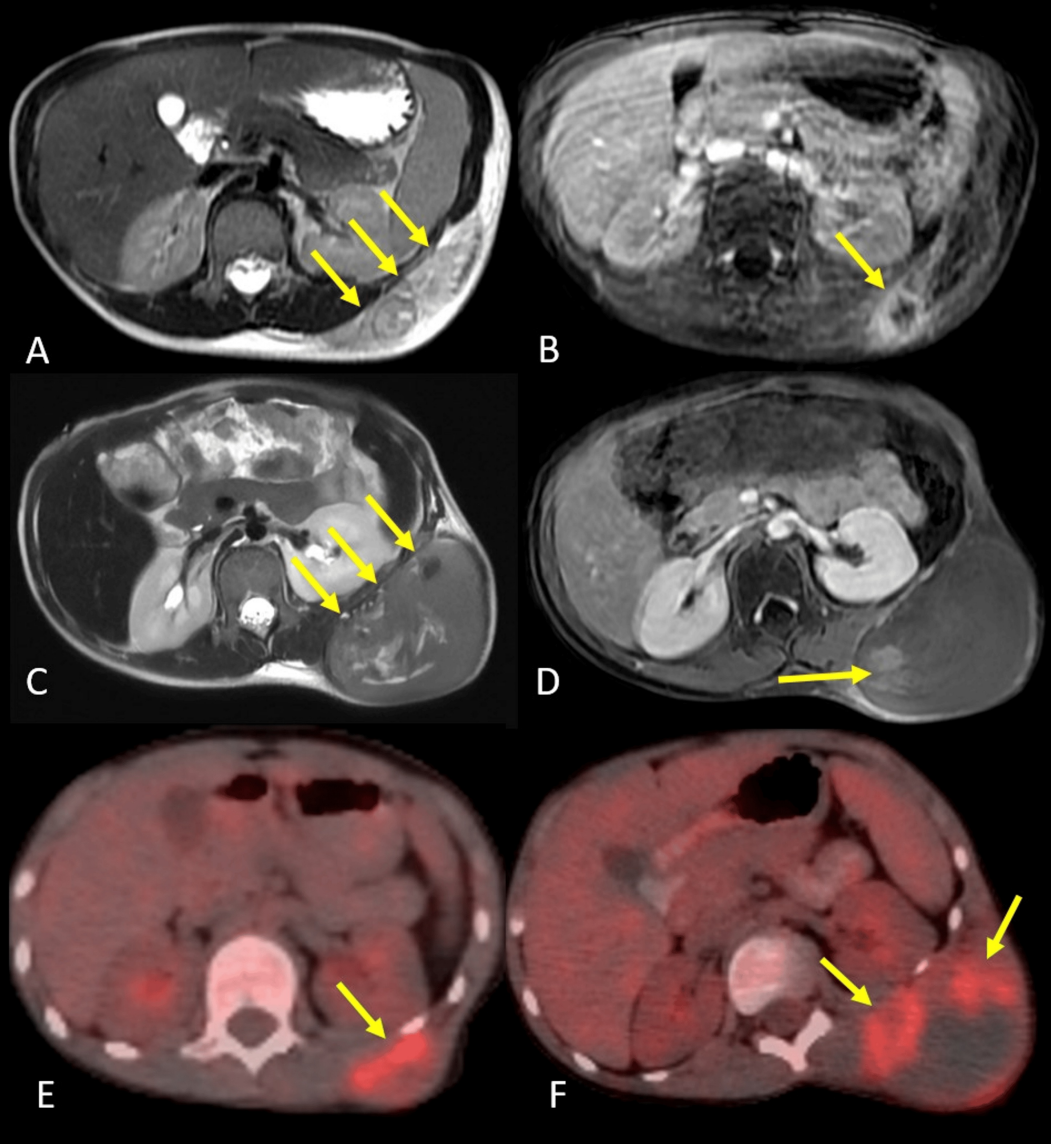
Baseline and follow-up imaging findings. At the time of diagnosis, (A) axial T2-weighted MR image shows a heterogeneous mass (arrows) in the subcutaneous fat tissue, and (B) axial postcontrast T1-weighted MR image shows a contrast-enhancing nodular component (arrow) within the mass. After induction chemotherapy, (C) axial T2-weighted MR image shows progression of the mass (arrows), and (D) axial postcontrast T1-weighted MR image shows a contrast-enhancing component (arrow) within it. Comparative PET-CT scans taken (E) at the time of diagnosis and (F) after induction chemotherapy, before RT, show an increase in mass size and FDG uptake (arrows). MR, magnetic resonance; PET-CT, positron emission tomography-computed tomography; FDG, fluorodeoxyglucose; RT, radiotherapy

Magnetic resonance imaging (MRI) revealed a 130x100x30 mm heterogeneous mass in the subcutaneous fat tissue with a contrast-enhancing nodular component (Figure 1A, 1B). The lesion was located at the level of the 11th and 12th ribs, extending into the adjacent intercostal spaces, without evidence of bone destruction or intrathoracic or intra-abdominal involvement, with mild uptake at the primary site and no distant metastases on F-18 Fluorodeoxyglucose (FDG) positron emission tomography (PET)-computed tomography (CT) scan.

**Figure 2:**
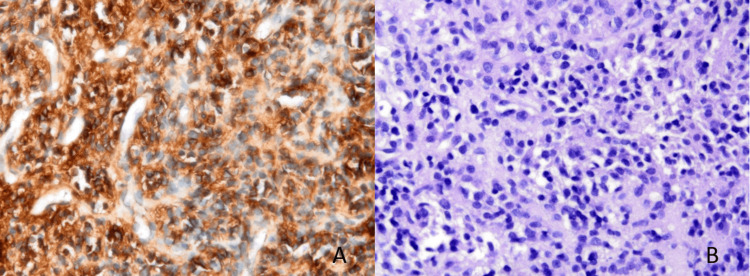
Histological and immunohistochemical characteristics of the tumor in the tru-cut biopsy specimen. A. Immunohistochemical staining reveals diffuse membranous positivity for CD99 in tumor cells (×460). B. Hematoxylin and eosin staining shows infiltration of small round blue cells (×460).

A tru-cut biopsy was performed, and the histopathological diagnosis was reported as EWS. In immunohistochemical studies, neoplastic cells were focally positive for CD99 and desmin and negative for S100, SMA, myogenin, myoD1, and EMA. There was noloss of H3K27me. EWSR translocation was demonstrated by the fluorescence in situ hybridization (FISH) procedure. These features are demonstrated in Figure 2.

Following the diagnosis of E-EWS, the patient was initiated on multi-agent chemotherapy in accordance with the Euro Ewing 2012 protocol, arm A-VIDE regimen (vincristine, ifosfamide, adriamycin, etoposide) for induction. After six cycles of induction chemotherapy, she was hospitalized due to febrile neutropenia, and progression of the tumor was seen both clinically and radiologically on the MRI scan taken for response assessment during the hospitalization period (Figure 1C, 1D). PET-CT was performed to exclude systemic progression after MRI demonstrated disease progression, and no metastasis was identified. Baseline and follow-up (after induction chemotherapy) FDG PET-CT images are shown in Figure 1E, 1F.

**Figure 3:**
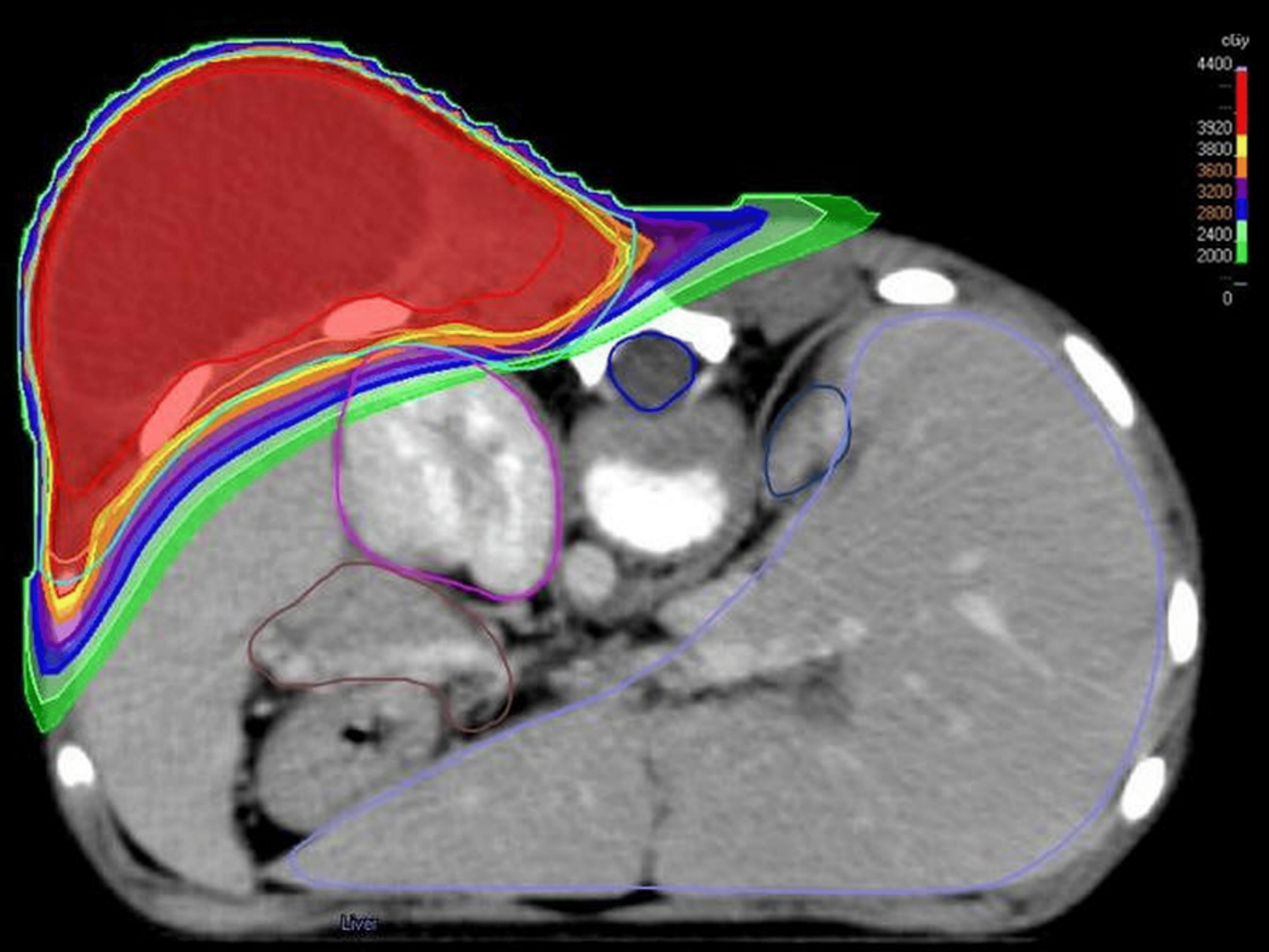
Dose color wash demonstrating the isodose distribution in the RT plan. RT, radiotherapy

The patient was discussed in the pediatric tumor board, and it was predicted that R0 resection was not possible and that surgical intervention would create a significant tissue defect due to the tumor size. Preoperative RT was planned; she was positioned prone for CT simulation. Given the rapid progression of the tumor under chemotherapy, its large size, and the possibility of inoperability, an ablative dose of SBRT was planned. A total of 40 Gy in 5 fractions was prescribed to the clinical target volume, created by adding a 1 cm margin to the gross tumor volume (GTV), with some modifications in certain areas due to organs at risk (OARs), followed by an additional 5 mm expansion to create the planning target volume (PTV) [11]. A volume-based prescription was used, with the prescribed dose covering 95% of the PTV (D95), aiming for a homogeneous dose distribution within the target. The treatment, which was applied every other day, was completed in approximately two weeks. The calculated doses to OARs are given in Table 1, and the dose color wash is seen in Figure 3.

**Table 1:** Organ-at-risk doses for our patient treated with 40 Gy in 5 fractions.

Organ	Dose Constraint
Ipsilateral Kidney	Average Dose: 966 cGy
Ipsilateral Kidney	V23 Gy: 9.54 cm^3^
Contralateral Kidney	Average Dose: 365 cGy
Liver	Average Dose: 252 cGy
Spinal Cord	Dmax: 2045 cGy
Bowel Bag	Dmax: 3436 cGy
Bowel Bag	V24 Gy: 1.99 cm^3^

**Figure 4:**
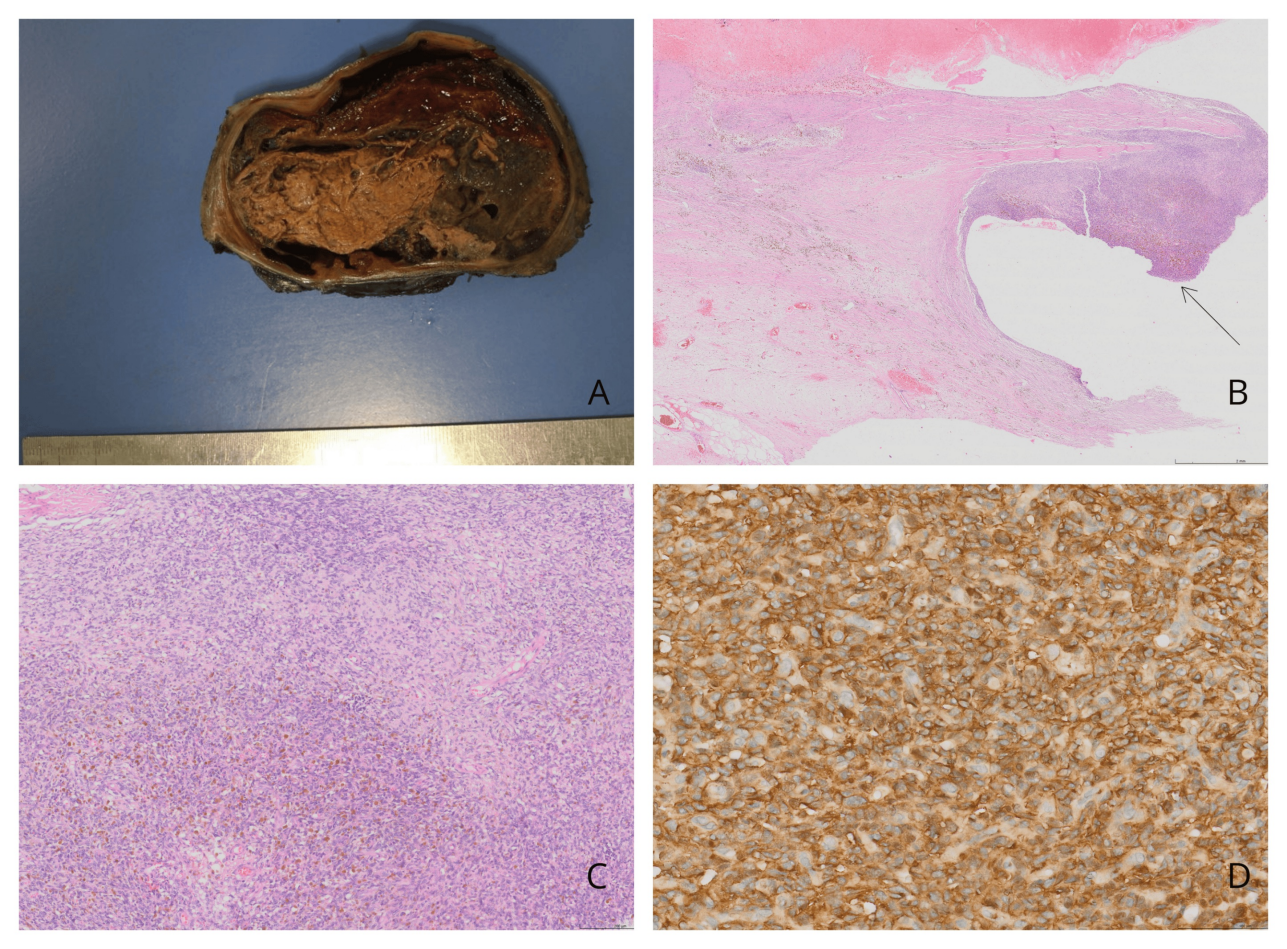
Histopathological and immunohistochemical findings of the postoperative resection specimen. A. Subcutaneous mass (14x6.5x5 cm) with a mostly necrotic and hemorrhagic cut surface. B. Large areas with histological findings of chemotherapy effect and viable tumor cells in a small area (arrow) (hematoxylin and eosin, scale bar: 2 mm). C. Tumor cells with a small round shape and scant cytoplasm in a solid pattern, with hemosiderin-laden macrophages between tumor cells (hematoxylin and eosin, scale bar: 200 µm). D. Diffuse cytoplasmic staining of tumor cells with CD99 (scale bar: 100 µm).

SBRT was well tolerated, with no acute toxicity observed other than mild dermatitis. End-of-treatment physical examination revealed softening of the mass but no significant decrease in size. The patient underwent surgery five weeks after treatment. During surgery, tumor invasion into the serratus muscles was seen; complete resection was performed by separating it with sharp and blunt dissection. The histopathological diagnosis was compatible with EWS (Figure 4), with secondary treatment-related changes and fewer than 1% viable tumor cells. No viable tumor cells were observed at the surgical margins.

**Figure 5:**
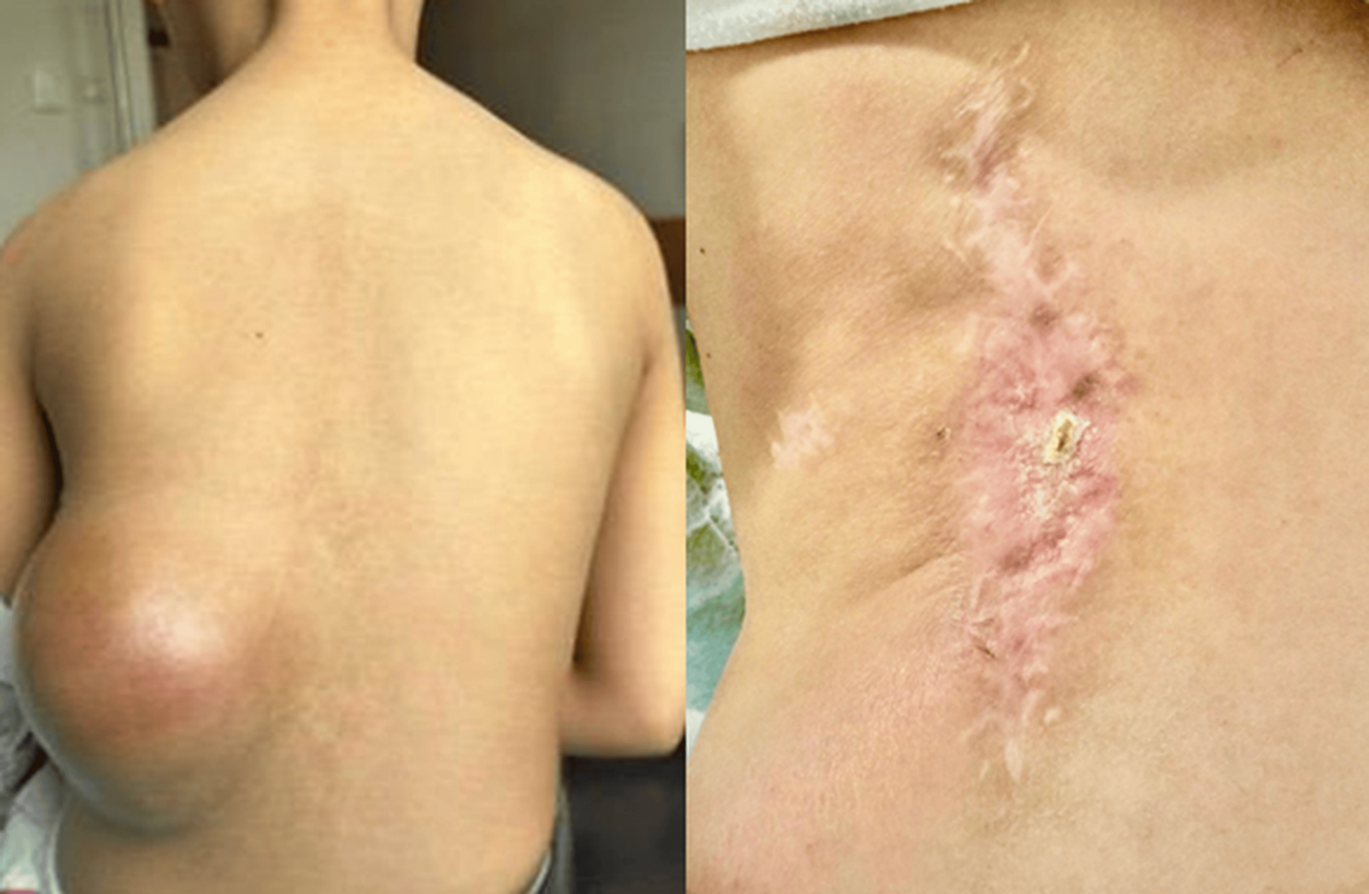
Clinical photographs before radiotherapy and at 14-month follow-up after SBRT. SBRT, stereotactic body radiotherapy

Postoperatively, wound healing complications were observed. According to the Common Terminology Criteria for Adverse Events (CTCAE), version 5.0, this was classified as a grade 3 wound complication requiring surgical revision of the operative site six months later. However, the wound site currently appears well healed (Figure 5).

After surgical recovery (six weeks later), consolidation chemotherapy was started in accordance with the Euro Ewing 2012 protocol arm A-VAC regimen (vincristine, actinomycin D, cyclophosphamide), and a total of six cycles were administered. Since the cessation of systemic therapy, she has remained recurrence-free for 18 months (corresponding to 24 months after SBRT).

## Discussion

This report underscores the impact of preoperative SBRT in a pediatric patient with E-EWS, demonstrating a near-complete pathological response despite isolated local tumor progression during induction chemotherapy.

Due to its rarity, no randomized prospective trials specifically address E-EWS. As a result, treatment approaches are primarily based on studies of skeletal EWS, as E-EWS is part of the EWS family of tumors and shares molecular and biological features with osseous EWS. In line with this, current treatment protocols generally do not differentiate between these types and recommend similar strategies for both [3,4].

According to current guidelines, preoperative RT should be considered in EWS patients with inadequate response to chemotherapy, especially if surgery carries substantial risk near critical structures [5]. Individual evaluation is recommended, as surgery may become possible with preoperative RT.

In studies such as the Cooperative Ewing's Sarcoma Study (CESS) 81, CESS 86, and the European Intergroup Cooperative Ewing's Sarcoma Study (EICESS) trials [12], 298 patients received postoperative RT and 240 received preoperative RT. LC was similar regardless of timing, but preoperative RT patients had more systemic failure, with poorer survival in EICESS-92 (58% vs. 71%). These outcomes may reflect selection bias, delayed tumor resection, or treatment interruptions due to infections. Note that these are studies from the 1900s that used outdated RT technologies.

There have been groundbreaking advances in RT technology over the last two decades. One of these advances is the SBRT technique, which provides high conformality and allows for high fraction dose delivery in 1 to 5 fractions. SBRT can provide better LC in relatively radioresistant tumors such as sarcomas [13]. Its steep dose fall-off offers better normal tissue sparing than conventional RT, which is particularly essential for late toxicities and chronic morbidities [10]. Furthermore, the rapid completion of SBRT allows these patients to receive systemic therapy or surgical interventions as soon as possible.

Although SBRT use in pediatric and young adult patients has increased, data on its safety and efficacy remain limited and heterogeneous regarding tumor types, treatment goals, and schedules [9,11]. It is often an alternative to surgery for oligometastatic or recurrent disease. The National Ewing Sarcoma Tumor Board recommends SBRT for consolidation as an effective alternative to fractionated RT for non-pulmonary metastases but does not advise it for primary EWS tumors [14].

Preoperative RT is recommended for patients with “expected marginal resections,” classically with a daily fraction dose of 1.8-2 Gy and a total dose ranging from 45 to 54 Gy, taking into account the tumor location and its proximity to critical structures [15]. However, the recently published Children's Oncology Group (COG) AEWS1031 trial evaluated the results of preoperative RT in localized EWS and found that the use of low-dose preoperative RT resulted in a high rate of R0 resection and excellent pathological response [7]. In this trial, 36 Gy preoperative RT was performed with a 1 cm margin to the GTV, starting with the beginning of consolidation chemotherapy, with or without 19.8 Gy boost, depending on the surgical margin status. In this study, eight of the 108 patients who received combined modality therapy received preoperative RT. R0 resection was achieved in seven of these eight cases; histopathologically, five had ≤1% viable tumor, and six had ≤5% viable tumor. With a median EFS of 55 months, no isolated local recurrence was seen; three-year EFS and OS rates were 62.5% and 87.5%, respectively. Grade 3 wound complications were observed in two cases.

The tumor's large size and rapid growth led us to choose a hypofractionated scheme. Given the low likelihood of surgery, SBRT was given with a 40 Gy dose in 5 fractions. The optimal SBRT dose in pediatric cases is unclear; the common schedule in the literature is 6-8 Gy in 5 fractions [11]. Dose varies based on treatment aim, lesion location, prior RT, and histology. A meta-analysis of nine studies applied SABR to 217 lesions in 142 patients, mostly with metastatic or recurrent disease, including 30% with EWS. The median dose was 35 Gy in 5 fractions. In light of this trial’s findings, a higher biological equivalent dose (BED)10 can improve LC, with every 10 Gy increase in BED10 associated with a 5% improvement in two-year LC (p=0.02) in sarcoma-predominant cohorts [10].

Information on the timing of surgery after preoperative RT is limited in EWS. In soft tissue sarcomas, it is generally recommended to wait four to eight weeks after preoperative RT to reduce acute surgical complications [16]. In the COG AEWS 1031 trial, consolidation chemotherapy was administered concurrently with preoperative RT during the first four weeks of consolidation, followed by surgery as soon as possible [7]. However, the time between RT and surgery was not specified in the study. On the other hand, delayed time to local therapy ≥16 weeks after chemotherapy initiation was associated with worse survival in EWS [17]. Because there was no significant shrinkage in the mass other than mild softening in our patient, surgery was planned approximately five weeks after SBRT to reduce wound complications.

In patients with EWS who underwent surgery, positive surgical margin status is one of the most important poor prognostic factors. According to the report from the CESS trials, the local or combined local and systemic relapses are high in cases with incomplete tumor resection (12% vs. 5%, P=0.045) [18]. Other known poor prognostic factors for OS and EFS in a non-metastatic setting include axial localization of the tumor, large tumor size (≥8 cm), and limited response to chemotherapy [19]. However, there is a lack of data in the literature regarding the importance of tumor response after preoperative RT. In our patient, who has remained disease-free for approximately 24 months, the achievement of R0 resection and a near-complete pathological response (<1% viable tumor cells) following preoperative SBRT may be associated with a favorable outcome.

Nonetheless, as previously underlined, wound complications are an important aspect and a major concern in RT given in the preoperative period. However, as we know from soft tissue sarcoma studies, using modern RT techniques such as intensity-modulated radiation therapy or volumetric modulated arc therapy has reduced wound-healing complications [20]. In the present case, wound healing problems persisted in the postoperative period and even six months later, requiring revision of the surgical bed, but the patient recovered completely during follow-up and had no sequelae.

## Conclusions

This report describes a pediatric E-EWS case with a large lesion that progressed despite initial chemotherapy, rendering complete tumor excision unfeasible. Through multidisciplinary management and the addition of preoperative RT, gross total resection was ultimately achieved, and she remains disease-free at 24 months of follow-up. We aim to highlight that SBRT may serve as a valuable adjunct in selected challenging cases, particularly in the pediatric population. Nevertheless, further prospective studies are warranted to establish the optimal dose, fractionation, and timing of SBRT in both preoperative and definitive settings.
